# Ginsenoside-Rg5 induces apoptosis and DNA damage in human cervical cancer cells

**DOI:** 10.3892/mmr.2014.2821

**Published:** 2014-10-30

**Authors:** LI-DAN LIANG, TAO HE, TING-WEI DU, YONG-GANG FAN, DIAN-SEN CHEN, YAN WANG

**Affiliations:** Department of Obstetrics and Gynecology, The First Affiliated Hospital of Henan University of Science and Technology, Luoyang, Henan 471003, P.R. China

**Keywords:** cervical cancer, ginsenoside-Rg5, DNA damage, apoptosis

## Abstract

*Panax ginseng* is traditionally used as a remedy for cancer, inflammation, stress and aging, and ginsenoside-Rg5 is a major bioactive constituent of steamed ginseng. The present study aimed to evaluate whether ginsenoside-Rg5 had any marked cytotoxic, apoptotic or DNA-damaging effects in human cervical cancer cells. Five human cervical cancer cell lines (HeLa, MS751, C33A, Me180 and HT-3) were used to investigate the cytotoxicity of ginsenoside-Rg5 using a 3-(4,5-dimethylthiazol-2-yl)-2,5-diphenyltetrazolium bromide assay. Additionally, the effects of ginsenoside-Rg5 on the apoptosis of HeLa and MS751 cells were detected using DNA ladder assays and flow cytometry. DNA damage was assessed in the HeLa and MS751 cells using alkaline comet assays and by detection of γH2AX focus formation. The HeLa and MS751 cells were significantly more sensitive to ginsenoside-Rg5 treatment compared with the C-33A, HT-3 and Me180 cells. As expected, ginsenoside-Rg5 induced significant concentration- and time-dependent increases in apoptosis. In addition, ginsenoside-Rg5 induced significant concentration-dependent increases in the level of DNA damage compared with the negative control. Consistent with the comet assay data, the percentage of γH2AX-positive HeLa and MS751 cells also revealed that ginsenoside-Rg5 caused DNA double-strands to break in a concentration-dependent manner. In conclusion, ginsenoside-Rg5 had marked genotoxic effects in the HeLa and MS751 cells and, thus, demonstrates potential as a genotoxic or cytotoxic drug for the treatment of cervical cancer.

## Introduction

Cervical cancer, which develops in the tissues on the surface of the cervix, is one of the most common types of cancer affecting females worldwide, with >510,000 new cases and 288,000 mortalities per year (1). The main risk factor for developing cervical cancer is contraction of human papillomavirus (HPV), which is the cause of almost all cases of cervical cancer (2). The introduction of HPV vaccines has afforded major advances in the prevention and management of cervical cancer; however, current vaccines can prevent only ~70% of cases (1). HPV infections are often asymptomatic and transient, but can promote *in situ* cervical pre-cancer, although the majority of cases spontaneously regress, indicating that HPV alone is not sufficient to cause cervical cancer. In addition, females in the early stages of cervical cancer may not exhibit any symptoms (2). Regular Pap tests are now more common, making cervical cancer more detectable (3), and the early detection of cervical cancer tends to lead to more favorable treatment outcomes compared with the detection of more advanced stages (4).

There are several treatment options for cervical cancer, including radiation and chemotherapy, which can be used in conjunction with various herbal remedies (5,6). While controversial in the medical field, herbal treatments can produce positive results when used alongside more standard therapies (7). Furthermore, traditional Chinese medicine (TCM) combined with a healthy lifestyle can often lead to the complete resolution of mild and moderate diseases (8). Several doctors have used the TCM ginseng ginsenoside-Rh2 in an attempt to further improve immune system function, inhibit cancer cell proliferation and induce the transformation of normal cells in patients with cervical cancer. Additionally, when used at a dose of 60 g/kg, sophora root has a significant effect on the development and progression of cervical cancer in mice (http://www.acupuncture.com/herbs/cancerherbc.htm. Accessed March 4, 2013).

TCM is a holistic medicinal system, which includes the use of herbal medicines, acupuncture and moxibustion, tuina, dietary therapy and qigong. TCM has specific methods for diagnosis and treatment, primarily associated with differentiation of the syndrome and the prescription of herbal formulas (9). A systematic review of case reports reveals that there is an abundance of support for the use of TCMs as therapy for a variety of types of cancer, suggesting the potential benefits of these therapies (10).

**Figure 1:**
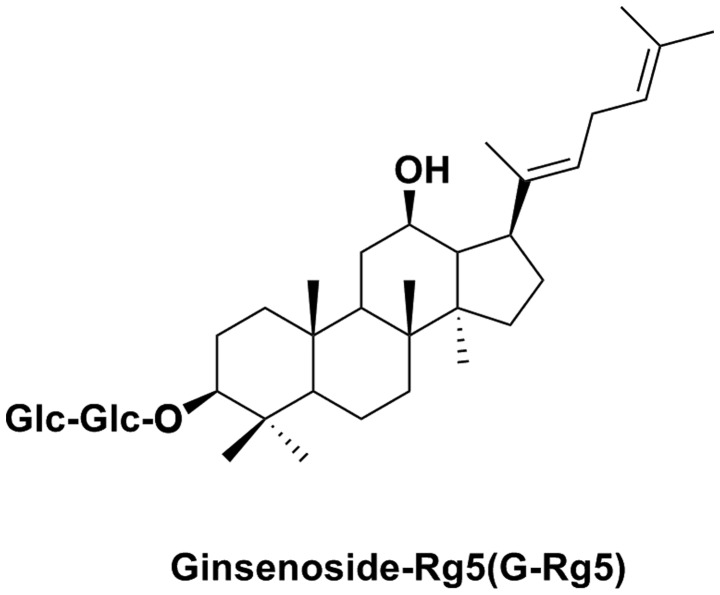
Chemical structure of ginsenoside-Rg5.

The use of ginseng as a TCM is common in the treatment of diabetes, cancer, stress and allergies in several Asian countries (11). In particular, heat-processed ginseng, which has been used for the treatment of cancer, inflammation and aging, contains ginsenoside-Rg5 (Fig. 1) as a main constituent (12–14); ginsenoside-Rg5 belongs to the family of protopanaxadiol ginsenosides (12,13) and has been demonstrated to exhibit marked anticancer activity (15,16), antidermatitic activity (17), anti-inflammatory effects in mouse lungs (18), neuroprotective effects (19) and microglial activation (20). However, the effects of ginsenoside-Rg5 on cervical cancer remain to be elucidated. During screening for the identification of TCMs that inhibit the progression of cervical cancer, heat-processed ginseng and its main constituent ginsenoside-Rg5 potently induced apoptosis and DNA damage in human cervical cancer cells *in vitro*. Therefore the present study investigated the applicability of ginsenoside-Rg5 as a potential cytotoxic or genotoxic drug for the treatment of cervical cancer.

## Materials and methods

### Cell lines

The human cervical cancer cell lines, C-33A (cat. no. HTB-31), HT-3 (cat. no. HTB-32), Me180 (cat. no. HTB-33) and MS751 (cat. no. HTB-34), were obtained from American Type Culture Collection (Manassas, VA, USA). HeLa cells, derived from human cervical carcinoma, were purchased from the Cell Bank of the Type Culture Collection of the Chinese Academy of Sciences (Shanghai, China). The HeLa cells were cultured in Dulbecco’s modified Eagle’s medium (DMEM) supplemented with 10% fetal bovine serum (FBS) and 1% streptomycin and penicillin, followed by incubation at 37°C in a 5% CO_2_ incubator. The C33A cells were cultured in DMEM containing l-glutamine, 10% FBS, 1% non-essential amino acids and 1% sodium pyruvate. The HT-3 and Me180 cells were cultured in McCoy’s medium (Sigma-Aldrich, St. Louis, MO, USA) with l-glutamine and 10% FBS. The MS751 cells were cultured in Eagle’s minimum essential medium (Sigma-Aldrich) with l-glutamine, 10% FBS, 1% non-essential amino acids and 1% sodium pyruvate.

### Chemicals and reagents

Ginsenoside-Rg5 (purity>95%) was provided by Dr WM Zhao at the Shanghai Institute of Materia Medica of the Chinese Academy of Sciences (Shanghai, China). Dimethylsulfoxide, Trypan blue, low melting agarose, 4′,6-diamidino-2-phenylindole (DAPI), normal melting agarose, 2-amino-2-(hydroxymethyl)-1,3-propanediol, sodium dodecyl sulfate, ethylenediaminetetraacetic acid, 3-(4,5-dimethylthiazol-2-yl)-2,5-diphenyltetrazolium bromide (MTT), N-methyl-N-nitro-N-nitrosoguanidine (MNNG, inducing DNA damage), Tween 20 and paraformaldehyde were obtained from Sigma-Aldrich. Propidium iodide (PI) (21) and DMEM were obtained from Gibco Life Technologies (Carlsbad, CA, USA). The Apoptosis Detection kit I was obtained from BD Pharmingen San Diego, CA, USA). Triton X-100, ethidium bromide, FBS, xylene cyanol and bromphenol blue were obtained from Shanghai Sangon Biotech Co., Ltd. (Shanghai, China).

### Cell viability assessment

The cells were seeded into 96-well plates at a density of 5×10^4^ cells per well and left overnight for cell adherence. Cell viability was determined between 12 and 48 h in the presence or absence of metformin using an MTT assay (22). Plates were read using an Automated Microplate Reader (Multiskan EX; Lab Systems, Helskinki, Finland) at a test wavelength of 570 nm.

### DNA extraction and the detection of DNA fragmentation

The HeLa and MS751 cells were treated using ginsenoside-Rg5 at concentrations of 0, 2.5 or 5 μM for 12 or 24 h, respectively. Cells treated with 10 μM MNNG for 24 h were used as a positive control (CT). The treated cells were then harvested and washed with phosphate-buffered saline (PBS). DNA was extracted using a Wizard Genomic DNA Purification kit (Promega Corporation, Madison, WI, USA) (23). DNA fragmentation was detected by electrophoresis on a denaturing urea polyacrylamide gel, which was stained using silver nitrate solution (24).

### Detection of apoptotic incidence using flow cytometry (25)

Cell apoptosis was determined using an Annexin V-fluorescein isothiocyanate (FITC) apoptosis detection kit I (BD Pharmingen). Briefly, the cells were treated with ginsenoside-Rg5 and MNNG at concentrations between 0 and 5 μM and at 10 μM, respectively, for 24 h. The cells were then washed twice with cold PBS and resuspended in 500 μl binding buffer at a concentration of 5×10^5^ cells/ml. Subsequently, 5 μl annexin V-FITC solution and 5 μl PI (1 mg/ml) were added to the cells at 37°C for 30 min. The cells were analyzed using flow cytometry within 1 h. The number of apoptotic cells were counted and represented as a percentage of the total cell count.

### Alkaline comet assay

The HeLa and MS751 cells were treated with 0, 0.625, 1.25, 2.5 or 5 *μ*M ginsenoside-Rg5 for 24 h and were then collected, washed and suspended in PBS (pH 7.4). Subsequently, 30 μl cell samples (1×10^4^ cells) were suspended in 110 μl of 1% molten low-melting-point agarose at 37°C (26). The monosuspension was cast on a microscopic slide that had been covered with a layer of 0.8% regular-melting-point agarose. Images were captured using an Olympus BX53 fluorescent microscope (Olympus Corporation, Tokyo, Japan) using a filter of 515–560 nm. The extent of DNA migration was determined using an image analysis system (CASPLab; www.casp.of.pl) and the tail length, indicating DNA migration from the nucleus, and tail moment (migrated DNA in the tail multiplied by the tail length) were recorded. All cells treated with MNNG (10 *μ*M) were included as positive controls.

### γH2AX foci staining

The phosphorylation of histone H2AX as a marker of DNA double-strand breaks (DSBs) was performed, as previously described (24). The HeLa and MS751 cells (5×10^5^) were seeded into six-well culture plates and treated with 0, 0.625, 1.25, 2.5 or 5 μM ginsenoside-Rg5 and 10 μM MNNG for 24 h. Following treatment, the cells were fixed in 4% paraformaldehyde for 15 min, washed with PBS (pH 7.4) and 0.1% Tween 20 and permeabilized in 1% Triton-X 100 for 30 min. Following inhibition with fetal bovine serum (Gibco Life Technologies, Carlsbad, CA, USA) for 60 min, the cells were incubated with rabbit monoclonal anti-gH2AX antibodies (1:1,500; Cell Signaling Technology, Inc., Danvers, MA, USA) overnight at 4°C and conjugated with Alexa594-conjugated anti-rabbit secondary antibodies (1:360, Cell Signaling Technology, Inc.) for 60 min. For nuclear staining, 1 mg/ml DAPI was added to the cells and the cells were incubated for 15 min. The cells were then mounted in antifade media and images were captured using an Olympus BX53 fluorescent microscope (Olympus Corporation). The objectives were set at wavelengths of 594 nm for γH2AX and 350 nm for DAPI.

### Statistical analysis

All values are expressed as the mean ± standard error of the mean. The data were analyzed using one-way analysis of variance followed by Student-Newman-Keuls test for multiple comparisons. The Newman-Keuls multiple comparisons test was applied, and P<0.05 and P<0.01 were considered to indicate statistically significant differences.

## Results

### Ginsenoside-Rg5 inhibits the growth of cervical cancer cells

**Figure 2:**
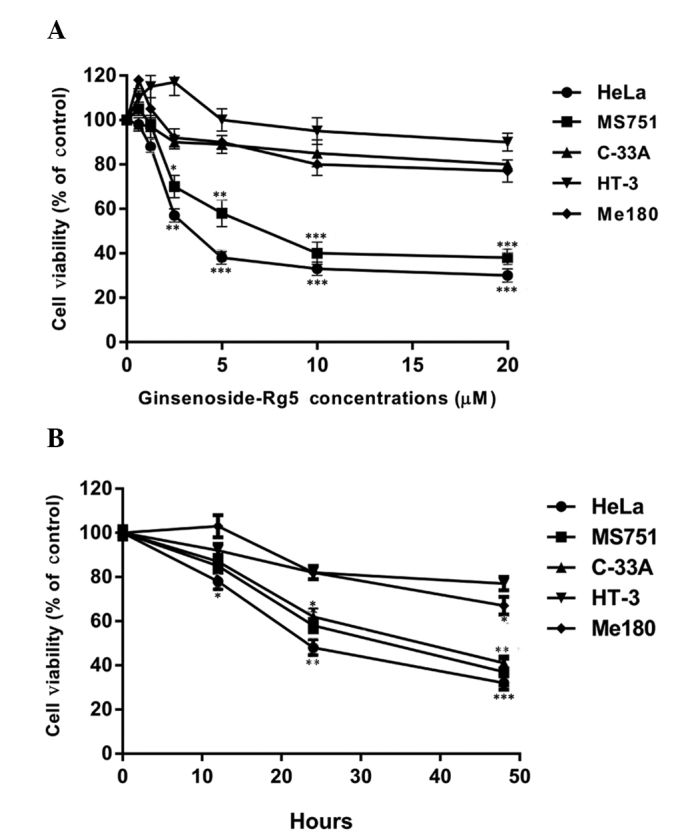
Ginsenoside-Rg5 inhibits the growth of cervical cancer cells. The human cervical cancer cell lines, HeLa, MS751, C33A, Me180 and HT-3, were cultured in medium as described in the Materials and methods. The cells were exposed to (A) 0, 0.625, 1.25, 2.5, 5, 10 or 20 μM ginsenoside-Rg5 for 48 h or (B) 5 μM ginsenoside-Rg5 for 0, 12, 24, or 48 h. Four hours prior to the final analysis, 100 μg/ml MTT was added to the cell culture medium and the cell viability was determined using an MTT assay. The number of cells in the untreated sample was set as 100%. Data are expressed as the mean ± standard deviation of three independent experiments.^*^P<0.05, ^**^P<0.01 and ^***^P<0.001, compared with the control. MTT, 3-(4,5-dimethylthiazol-2-yl)-2,5-diphenyltetrazolium bromide.

To determine whether ginsenoside-Rg5 exerted cytotoxic effects on the cervical cancer cells, the cervical cancer cell lines were exposed to various concentrations of ginsenoside-Rg5 and the cell viability was assessed. Although the sensitivities to the treatment varied depending on the cell line, ginsenoside-Rg5 treatment at concentrations ranging from 0.625 to 20 μM for 48 h resulted in concentration-dependent cytotoxicity in the cervical cancer cells (Fig. 2A). The half-maximal inhibitory concentration (IC_50_), the concentration at which 50% of the cells survive, values of the HeLa and MS751 cells were between 2.5 and 10 μM, while the IC_50_ values of the C-33A, HT-3 and Me180 cells were all higher than 20 μM (Fig. 2A). To further validate the effects of ginsenoside-Rg5, the cells were exposed to 5 μM ginsenoside-Rg5 for 12, 24 or 48 h. Ginsenoside-Rg5 reduced the viability of the cervical cancer cells in a time-dependent manner (Fig. 2B). The HeLa and MS751 cells had a higher sensitivity to the treatment compared with the C-33A, HT-3 and Me180 cells. Thus, these results suggested that ginsenoside-Rg5 possessed a cytotoxic function in certain cervical cancer cell lines, including HeLa and MS751.

### Ginsenoside-Rg5-induces apoptosis in HeLa and MS751 cells

**Figure 3:**
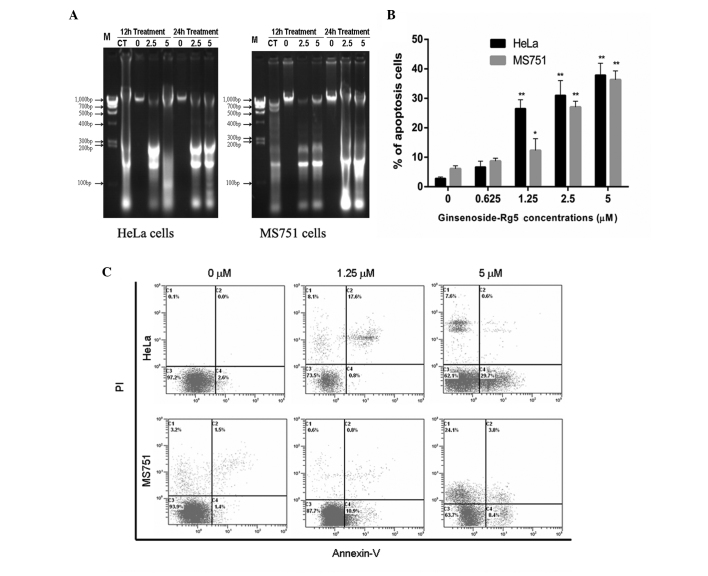
Ginsenoside-Rg5 induces apoptosis in HeLa and MS751 cells. (A) Apoptosis-associated DNA fragmentation in HeLa and MS751 cells. (B) Apoptotic rates of HeLa and MS751 cells treated with ginsenoside-Rg5 (0–5 μM) and MNNG (10 μM) for 24 h. Data are expressed as the mean ± standard error of the mean of three independent experiments. ^*^P<0.05 and ^**^P<0.01, vs. negative control (0 μM ginsenoside-Rg5). P-values refer to the comparison of baseline independent characteristics. (C) Flow cytometric analysis of apoptosis using Annexin-V and PI double-staining. MNNG, N-methyl-N-nitro-N-nitrosoguanidin; M, marker of DNA ladder; CT, positive control (10 μM MNNG); PI, propidium iodide.

The HeLa and MS751 cells were significantly more sensitive to ginsenoside-Rg5. Therefore, to determine whether the loss in cell viability induced by ginsenoside-Rg5 was associated with apoptosis, ginsenoside-Rg5-induced apoptosis was examined in these sensitive cell lines using DNA fragmentation analysis and flow cytometry. As shown in Fig. 3A, ginsenoside-Rg5 induced a ladder-like pattern on the urea polyacrylamide gel electrophoresis (PAGE). The DNA in the untreated cells remained intact. At concentrations of 2.5 and 5 μM, ginsenoside-Rg5 led to concentration- and time-dependent increases in DNA fragmentation. In addition, in HeLa and MS751 cells, the fraction of apoptotic cells increased markedly when the cells were treated with 1.25–5 μM ginsenoside-Rg5 (Fig. 3B). Taken together, these results suggested that the anticancer activity of ginsenoside-Rg5 was mediated, in part, by the induction of apoptosis.

### Ginsenoside-Rg5 induces DNA damage in HeLa and MS751 cells

**Figure 4:**
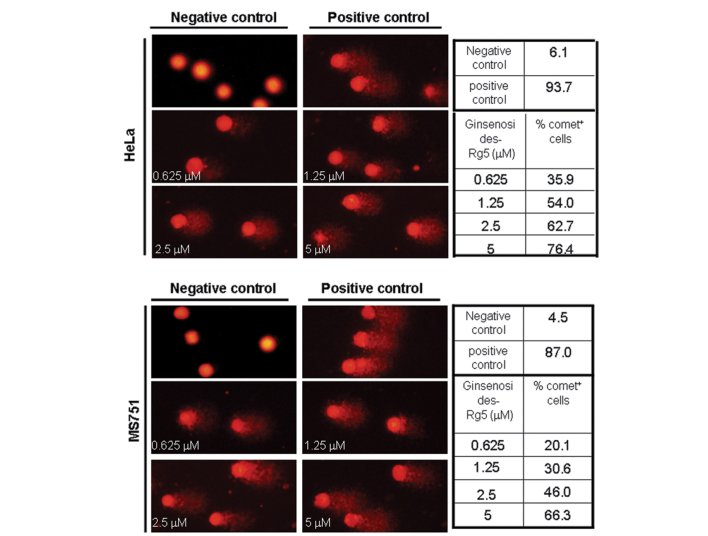
Comet images (magnification, ×200) of alkaline gel electrophoresis demonstrating DNA damage in the HeLa and MS751 cells. Treatment with 10 μM N-methyl-N-nitro-N-nitrosoguanidin was used as a positive control and 300 cells were counted at each concentration.

DNA strand breaks were induced by ginsenoside-Rg5, as demonstrated by the comet assays. The alkaline comet assay revealed that DNA fragments migrated to form comet-like images indicative of DNA damage (27). In the control cells, high-density DNA was observed in the comet heads with smooth margins and intact nuclei. The comet frequencies in the HeLa and MS751 cells were 6.1 and 4.5%, respectively. In the cells treated with ginsenoside-Rg5, the DNA comets exhibited broom-shaped tails. The percentages of comet-positive HeLa and MS751 cells significantly increased at concentrations between 0 and 5 μM compared with the negative control (Fig. 4).

The mean ± SEM of the tail length and tail moment in the HeLa and MS751 cells are shown in Table I. These results indicated that the cells, which were exposed to different concentrations of ginsenoside-Rg5, exhibited significantly higher DNA damage (P<0.01) compared with the control samples. In these cell lines, ginsenoside-Rg5 significantly increased the tail length (P<0.01) and tail moment (P<0.01) when used at concentrations of 1.25 to 5 μM.

### γH2AX foci reveal the induction of DNA DSBs by ginsenoside-Rg5

**Figure 5:**
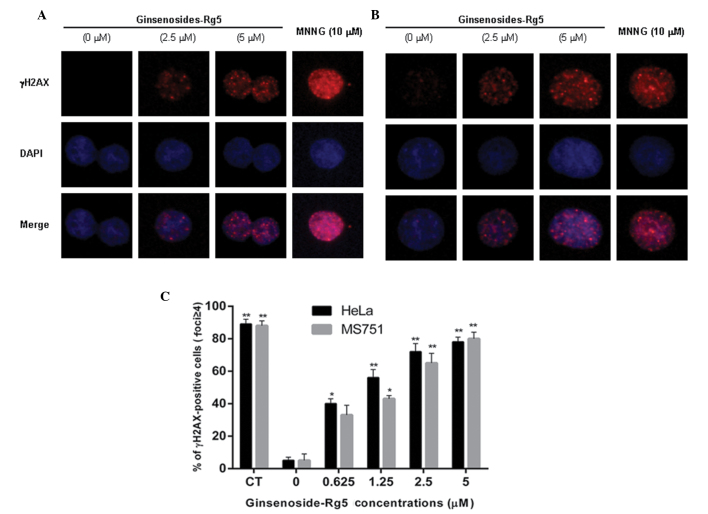
DNA double-strand breaks were visualized using γH2AX foci formation in the HeLa and MS751 cells. (A) Images of γH2AX foci formation in cells. Anti-γH2AX monoclonal antibodies were used to detect DNA damage foci immunofluorescence and DAPI was used for nuclei staining (magnification, ×400). (B) Percentages of γH2AX-positive cells were measured following treatment with 10 μM MNNG (CT) and 0, 0.625, 1.25, 2.5 or 5 μM ginsenoside-Rg5 for 24 h. Data are expressed as the mean ± standard error of the mean of three independent experiments. ^*^P<0.05 and ^**^P<0.01, compared with the negative control. CT, positive control; MNNG, N-methyl-N-nitro-N-nitrosoguanidin; DAPI, 4′,6-diamidino-2-phenylindole.

The phosphorylation of histone H2AX foci formation has been suggested as a sensitive way to detect DNA DSBs (27). A threshold of four or more γH2AX foci/cell is optimal for determining the extent of DNA damage (28). Immunofluorescent images of histone H2AX phosphorylation in the γH2AX-stained HeLa and MS751 cells are shown in Fig. 5A. Ginsenoside-Rg5 caused a concentration-dependent induction of γH2AX foci. In the control, the HeLa and MS751 cells had few γH2AX-positive foci in the nuclei and there were ~5.5% cells containing over four foci (Fig. 5B). All treatments with ginsenoside-Rg5 and MNNG induced the formation of foci and increased the percentages of γH2AX-positive cells. In addition, ginsenoside-Rg5 and MNNG exhibited distinct concentration-dependent effects (P<0.01) on the formation of γH2AX foci in the HeLa and MS751 cells (Fig. 5B).

## Discussion

Conventional chemotherapeutic agents are often toxic to tumor cells and normal cells, which limits their therapeutic use. The identification of anticancer compounds from natural products offers a promising alternative to the use of synthetic compounds due to their favored safety and efficacy (29). Several preclinical and clinical studies have demonstrated the anticancer potential of *Panax ginseng*, which has been used clinically in China for thousands of years (19). In addition, ginseng is used to combat stress, fatigue, oxidizing agents, cancer and diabetes mellitus (30). The antitumor efficacy of ginseng is attributed mainly to the presence of saponins, also termed ginsenosides, which induce cell death and metastasis. Over 30 ginsenosides, which are triterpene derivatives, have been isolated from the ginseng saponin fraction and the chemical structures of the individual ginsenosides have been identified (15). Several types of eukaryotic cell death, including apoptosis, autophagy, paraptosis, mitotic catastrophe and necrosis have been recognized (31–33), of which apoptosis is the most common mechanism of cell death. Different types of ginsenoside induce apoptosis through a variety of signaling cascades. Ginsenoside-Rh2 and ginsenoside-Rg3 induce apoptosis in A549 lung adenocarcinoma cells, prostate cancer cells (34), hepatoma cells (35) and colorectal cancer cells (36). The induction of apoptosis by other ginsenosides, including Rb2, Rc and RS4, in different types of human tumor cell lines has also been observed (37,38). These findings suggest that the induction of tumor cell apoptosis by ginsenosides may be one of the mechanisms through which these compounds exert their antitumor effects. However, only a few studies have investigated the effects of ginsenosides and, in particular, ginsenoside-Rg5 in human cervical cancer.

Apoptosis, or programmed cell death, is the result of a highly complex cascade of cellular events that result in chromatin condensation and DNA fragmentation (39). It is a fundamental cellular event during development and is essential for radiation or drug-induced cytotoxicity, which is characterized by the cleavage of chromatin DNA into internucleosomal fragments. In the present study, denaturing urea PAGE was used to detect DNA fragmentation and the rate of apoptosis was measured using flow cytometry by double-labeling with annexin V and PI. The data demonstrated that ginsenoside-Rg5 caused concentration- and time-dependent increases in DNA fragmentation.

In addition, the majority of evidence has indicated that ginsenosides induce cell cycle arrest and apoptosis in mammalian tumor cells (35,40). However, few studies have described the genotoxicity of ginsenosides. Thus, the present study investigated the induction of DNA damage by ginsenoside-Rg5 in human cervical cancer cell lines *in vitro* by using alkaline comet assays and measuring γH2AX foci formation. The alkaline comet assay, a single-cell gel electrophoresis, is a method used to detect DNA strand breaks (41). Additionally, the formation of DNA DSBs induces γH2AX aggregation in the nucleus, thus, the measurement of γH2AX foci formation may be a sensitive method for the detection of DNA DSBs (42). In the present study, these assays revealed concentration-dependent increases in DNA damage, as evidenced by an increase in comet tail sizes with a concomitant reduction in head sizes in the comet assay, as well as an increased number of γH2AX-positive cells in response to ginsenoside-Rg5 treatment. Thus, these results revealed that the presence of γH2AX foci may be indicative of DSBs, confirmed by the comet assay. These results were consistent with those observed in previous studies (16,17), indicating that ginsenoside-Rg5 has antiproliferative and apoptotic activities in cancer cells.

In conclusion, the present study demonstrated that ginsenoside-Rg5 was anticarcinogenic, inducing cell DNA damage and apoptosis *in vitro,* and the DNA damage induced by ginsenoside-Rg5 may be associated with apoptosis. The results of the present study confirmed the antitumor effects of ginsenoside-Rg5 and the potential of ginsenoside-Rg5 as an agent of chemotherapeutic activity in human cervical cancer cells.

## Figures and Tables

**Table I:** Parameters of DNA damage from the comet assays of HeLa and MS751 cells exposed to different concentrations of ginsenoside-Rg5.

		Comet assay parameters			
	
Treatment group		Tail length (μM)		Tail moment	
		
Cell line/compound	Concentration	HeLa	MS751	HeLa	MS751
Negative control	0 μM	8.15±2.58	5.45±3.42	1.54±0.42	5.12±1.71
Ginsenoside-Rg5	0.625 μM	16.46±4.41	21.07±3.51	8.31±1.34	15.09±3.22
	1.25 μM	30.01±5.21^a^	48.42±9.52^a^	11.42±3.52^a^	25.66±3.16^a^
	2.5 μM	39.70±7.44^a^	57.48±8.87^a^	19.33±1.72^a^	33.76±4.21^a^
	5 μM	56.24±10.47^a^	72.06±4.70^a^	28.25±2.46^a^	36.31±2.99^a^
Positive control	10 μM	87.50±4.23^a^	80.08±2.82^a^	35.27±3.34^a^	43.08±6.36^a^

Data are expressed as the mean ± standard error of the mean of tail length and tail moment of the HeLa and MS751 cells in the alkaline comet assay.

a P<0.01, vs. negative control (0 μM ginsenoside-Rg5). represent the significance of DNA damage. P-values refer to the comparison of baseline independent characteristics. Positive control, N-methyl-N-nitro-N-nitrosoguanidine; negative control, phosphate-buffered saline (0 μM ginsenoside-Rg5).
